# Synchronous squamous cell carcinomas of the glans penis and scrotum: A case report and literature review

**DOI:** 10.1016/j.eucr.2026.103464

**Published:** 2026-04-29

**Authors:** Abdoul-Karim Paré, Delphine Yé, Hassami Sawadogo, Brahima Kirakoya, Amidou Bako, Mohamed Simporé, Salle Joseph Ramdé, Sinaly Soaré, Clotaire Alexis Marie Kiemdiba Donega Yaméogo, Adama Ouattara

**Affiliations:** a Division of Urology, Souro Sanou University Teaching Hospital, Bobo-Dioulasso, Burkina Faso; b Department of Urology, Dedougou Regional Hospital, Dedougou, Burkina Faso; c Division of Urology, Yalgado Ouedraogo University Teaching Hospital, Ouagadougou, Burkina Faso

**Keywords:** Penile squamous cell carcinoma, Scrotal squamous cell carcinoma, Synchronous tumours, HIV infection

## Abstract

Squamous cell carcinomas (SCCs) of the male external genitalia are uncommon malignancies, with scrotal SCC being particularly rare. The synchronous occurrence of SCC involving both the glans penis and the scrotum represents an exceptional clinical entity with limited documentation in the published literature. We report the case of a 70-year-old uncircumcised man with a background of HIV infection on antiretroviral therapy and remote tobacco use, who presented with a rare synchronous dual-site genital SCC. The pathophysiological, diagnostic, and therapeutic implications of this presentation are discussed in light of current evidence.

## 1 Introduction

Squamous cell carcinoma of the penis is a rare malignancy in developed countries, with an incidence below 1 per 100,000 men, rising to 2–3 per 100,000 in certain regions of sub-Saharan Africa and South America.^1^ Scrotal SCC is even rarer, accounting for approximately 1.5 cases per million men per year in Western populations.^2^^,^^3^ The synchronous occurrence of these two distinct genital malignancies in the same patient is exceptionally infrequent and raises important questions regarding shared pathophysiological mechanisms, common risk factors, and optimal therapeutic strategy.

Established risk factors for penile SCC include the absence of neonatal circumcision, phimosis, human papillomavirus (HPV) infection, tobacco use, poor genital hygiene, and immunosuppression.^1^^,^^4^ Scrotal SCC shares several of these risk factors. A recent systematic review on scrotal SCC highlighted that contemporary risk factors — principally immunosuppression and HPV infection — have largely supplanted the historical occupational carcinogen exposures previously associated with this tumour.^3^

HIV-related immunosuppression constitutes a particularly significant risk factor for the development of HPV-associated neoplasms, including both penile and scrotal SCC.^3^^,^^5^ We present a rare case of synchronous SCC of the glans penis and scrotum, and discuss the diagnostic and therapeutic implications of this clinical presentation.

## 2 Case presentation

G.A., a 70-year-old man with a known history of arterial hypertension and HIV infection managed with antiretroviral therapy (ART) for 20 years, along with remote tobacco cessation (20 years prior) and alcohol cessation (6 years prior), presented to our outpatient urology clinic with an eight-year history of a progressively enlarging ulcerated lesion of the right hemiscrotum. The lesion had evolved into an ulcero-proliferative mass. The patient reported multiple prior treatments of undocumented nature with no clinical improvement. He also complained of dysuria of approximately two years' duration, with no other associated urinary symptoms.

**Fig. 1:**
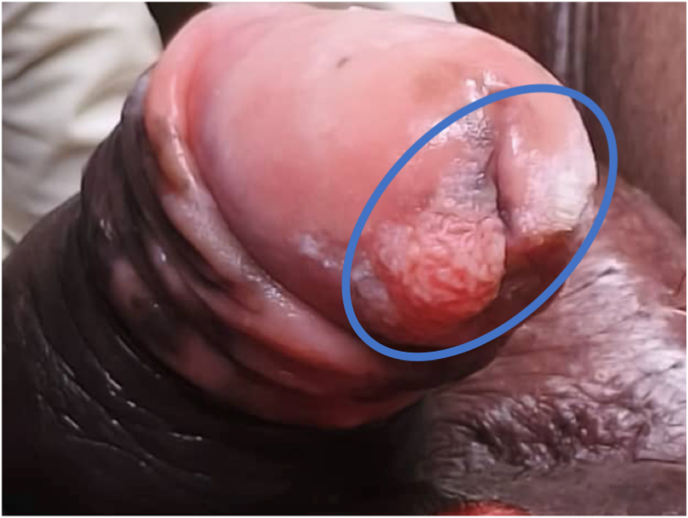
Proliferative peri-meatal lesion of the glans penis (delineated in blue).

On physical examination, the patient was uncircumcised and exhibited a reduced urinary stream on voiding observation. The prepuce appeared inflamed; upon retraction, a proliferative peri-meatal lesion was identified (Fig. 1).

**Fig. 2:**
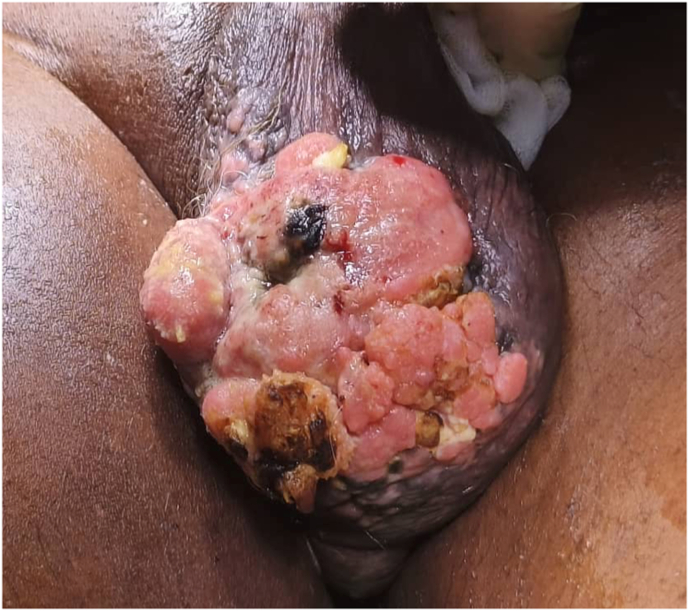
Ulcero-proliferative lesion of the right hemiscrotum.

The right hemiscrotum was swollen and harboured an ulcero-proliferative mass measuring approximately 7 cm in its greatest diameter, indurated and tender on palpation (Fig. 2).

The contralateral hemiscrotum was unremarkable on inspection and palpation. Digital rectal examination revealed a prostate of normal volume with no features suggestive of malignancy. No palpable lymphadenopathy was detected in the inguinal or supraclavicular regions. The remainder of the physical examination was unremarkable.

**Fig. 3:**
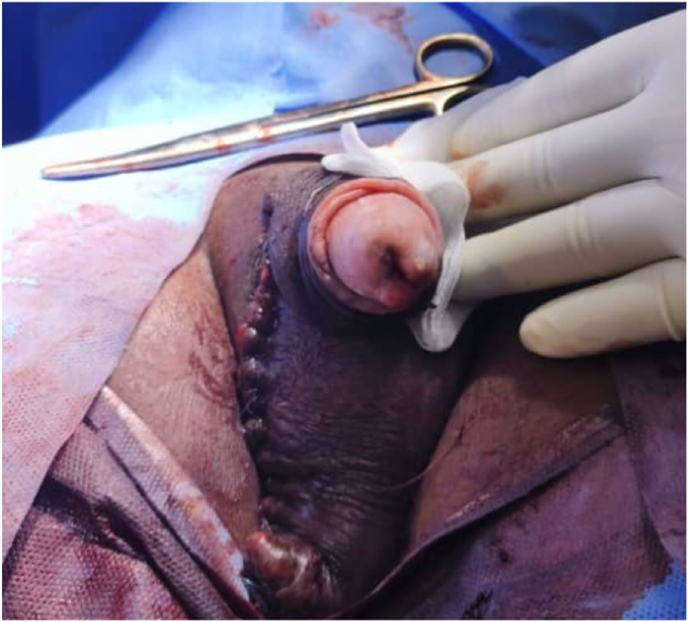
Operative view following biopsy of the glanular lesion and excision of the right scrotal mass.

Standard laboratory investigations, including a full blood count and assessment of renal and hepatic function, were within normal limits. Serum prostate-specific antigen (PSA) was 3.7 ng/mL. Immunovirological evaluation demonstrated a preserved CD4 T-lymphocyte count and an undetectable HIV viral load on current ART. A biopsy of the glanular lesion was performed. Given the chronic evolution of the scrotal mass (8 years) which resisted multiple previous undocumented treatments and its localized appearance which allowed for primary closure, a excisional biopsy was performed during the same surgical procedure (Fig. 3).

Histopathological examination of both specimens revealed a concordant morphological pattern at each site: well-differentiated keratinising squamous cell carcinoma involving the scrotum and the glans penis. No lymphovascular emboli were identified, and the surgical margins of the scrotal excision specimen were clear.

Thoraco-abdomino-pelvic computed tomography (CT) performed for staging purposes demonstrated no evidence of nodal or visceral metastatic disease, and no visible tumour continuity between the two lesions. The scrotal carcinoma was staged as cT_3_N_0_M_0_.

Following multidisciplinary tumour board discussion, the patient underwent circumcision combined with wide local excision of the glans tumour and dynamic sentinel lymph node biopsy (DSLNB). Given that the scrotal lesion had already been excised with clear margins during the diagnostic excisional biopsy, no further scrotal re-excision was required. Final histopathological analysis of the penile excision specimen confirmed well-differentiated SCC of the glans with invasion of the corpus spongiosum but without urethral involvement (pT_2_). All surgical margins were tumour-free. Sentinel lymph node histology was negative for malignancy.

**Fig. 4:**
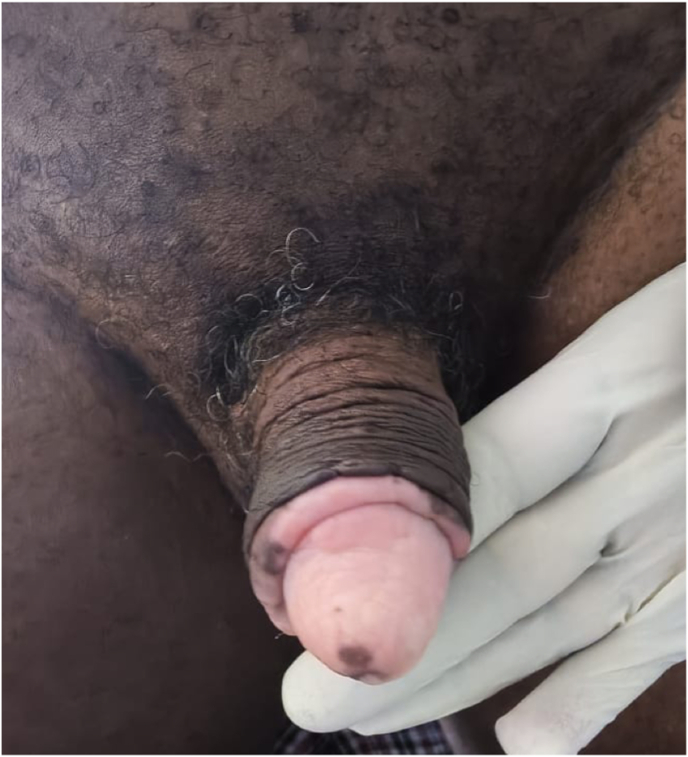
Appearance of the glans after one year of follow-up.

**Fig. 5:**
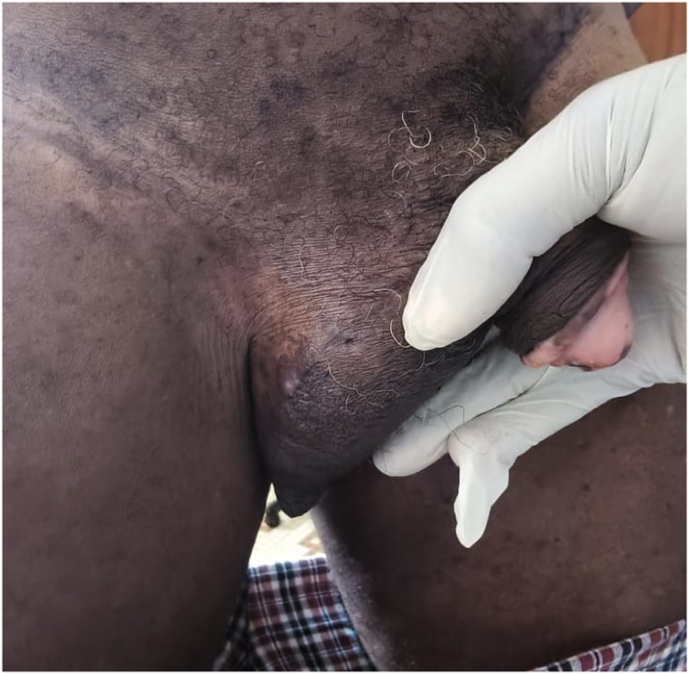
Scrotal appearance after one year of follow-up.

The postoperative course was uneventful, and the patient was discharged on postoperative day 3. At one-year follow-up, no tumour recurrence was detected at either the scrotal or glanular site (Fig. 4, Fig. 5). The patient reported no functional complaints.

## 3 Discussion

The synchronous occurrence of SCC involving both the glans penis and the scrotum appears to be uncommon and remains sparsely described in the literature. Consequently, data specifically addressing optimal management of this presentation are limited.

Our patient accumulated several well-established risk factors for genital SCC. The absence of circumcision promotes smegma accumulation and chronic local inflammation, creating a microenvironment conducive to carcinogenesis.^1^ HIV-related immunosuppression impairs anti-tumour immune surveillance and facilitates persistent HPV infection.^5^ Tobacco use — even remote, with cessation 20 years prior in this case — remains a significant independent risk factor for genital SCCs, with relative risk increases of three-to fivefold reported in the literature.^4^

The simultaneous presentation of neoplastic lesions at two anatomically distinct genital sites raises the possibility of multifocal HPV infection. High-risk HPV genotypes play a central role in male genital carcinogenesis.^6^ Among HPV-positive penile squamous cell carcinomas, HPV-16 is by far the predominant genotype, accounting for 68.3% of cases, followed by HPV-6 (8.1%) and HPV-18 (6.9%), while all other HPV types are detected much less frequently, according to a large systematic review and meta-analysis by Olesen et al..^7^ In immunocompromised patients, viral persistence is facilitated and the risk of malignant transformation is substantially elevated. The E6 and E7 oncoproteins of high-risk HPV strains inactivate the tumour suppressor proteins p53 and pRb, respectively, thereby driving uncontrolled cellular proliferation and the accumulation of oncogenic mutations.^6^ In the present case, the absence of HPV genotyping precluded confirmation of a viral etiology or determination of whether the two lesions resulted from independent infections or from a single disseminated strain.

The concept of “field cancerisation,” originally proposed by Slaughter et al., may equally apply: chronic exposure to shared carcinogens induces a genetically altered epithelial field that predisposes to the development of multiple independent neoplasms across a wide anatomical territory.^8^

Multifocal HPV-associated anogenital squamous cell carcinoma has been reported in HIV-positive patients. In a case series, three HIV-positive men with chronic condyloma acuminata developed multifocal anogenital SCC involving penile and perianal/perineal sites in the setting of impaired immune surveillance.^9^ Our patient shares the background of HIV infection and the presence of multiple genital tumours, but differs in that he presented with two anatomically distinct, synchronous primary SCCs of the glans penis and scrotum, with no history of preceding condyloma.

The diagnosis of genital SCCs rests on clinical assessment and histopathological confirmation by biopsy. Macroscopic appearance can vary considerably, ranging from exophytic papillary lesions to deeply infiltrative ulcerations, as observed in our patient.^1^ The co-occurrence of glanular and scrotal neoplastic lesions raises the critical differential diagnostic question of distinguishing between: (i) independent synchronous primary tumours, (ii) scrotal metastasis from a penile primary, or (iii) contiguous direct local extension.

Several arguments support the diagnosis of independent synchronous primary tumours rather than metastatic dissemination. First, the clinical timeline is inconsistent with metastatic disease: the scrotal lesion had been evolving for eight years, whereas urinary symptoms attributable to the glanular lesion appeared only two years prior — a chronology atypical for scrotal skin metastasis, which would logically follow, not precede, the diagnosis of the penile primary. Second, the absence of tumour continuity on CT imaging excludes direct local extension, which would necessitate progressive infiltration of the corpus spongiosum, penile shaft skin, and perineal soft tissues with palpable intermediate nodules or carcinomatous lymphangitis — none of which were present in this case. Third, the well-differentiated histological grade of both tumours, the absence of lymphovascular invasion, and the lack of regional lymphadenopathy on imaging collectively render metastatic dissemination unlikely.

Surgical excision with histologically clear margins constitutes the standard of care for both penile and scrotal SCC.^2^^,^^10^ For glanular tumours, surgical options range from organ-sparing approaches (wide local excision, glans resurfacing, or glansectomy) to partial or total penectomy, depending on tumour stage and grade. Current guidelines no longer specify a numerical minimum margin distance; instead, they emphasise the achievement of histologically negative margins, ideally confirmed by intraoperative frozen section analysis. Wider margins are advised for aggressive histological subtypes.^10^ For scrotal SCC, wide local excision with or without reconstructive closure is generally sufficient in the absence of deep tissue involvement.

Lymph node management — a key prognostic determinant — is risk-stratified. In clinically node-negative patients (cN_0_), surgical nodal staging (preferably by DSLNB) is recommended for high-risk tumours (stage ≥ pT_1_b), while active surveillance may be offered for low-risk disease.^10^ In the present case, the proliferative and clinically infiltrative character of the glanular lesion prompted the decision to perform DSLNB.

Multimodal treatment strategies include neoadjuvant cisplatin- and taxane-based chemotherapy for advanced nodal disease (bulky cN_2_ or cN_3_), and adjuvant chemotherapy may be considered on an individual basis for very high-risk patients (pN_3_). Radiotherapy represents an organ-preserving alternative for selected T_1_–T_2_ tumours and may be employed in the adjuvant setting.^10^

Postoperative surveillance must be sustained and systematic: clinical assessment every three months during the first two years, followed by six-monthly reviews until year five.^10^

As limitations of the study, we mention the absence of HPV genotyping and p16 immunohistochemistry, which does not allow us to formally attribute these synchronous tumours to an HPV etiology, nor to determine whether they result from two independent infections or from the same disseminated strain. This reflects the diagnostic resource constraints of our institution.

## 4 Conclusion

Synchronous squamous cell carcinoma of the glans penis and scrotum represents an exceptional clinical presentation that raises important pathophysiological questions regarding the mechanisms of multifocal genital carcinogenesis. This case underscores the imperative for thorough examination of all external genitalia in patients presenting with any suspicious genital lesion, particularly in the context of immunosuppression. Management should be guided by surgical excision with clear margins, combined with rigorous assessment of regional lymph node status.

## CRediT authorship contribution statement

**Abdoul-Karim Paré:** Writing – review & editing, Writing – original draft, Validation, Supervision, Methodology, Conceptualization. **Delphine Yé:** Writing – review & editing, Validation, Methodology, Conceptualization. **Hassami Sawadogo:** Writing – review & editing, Writing – original draft, Validation, Methodology, Conceptualization. **Brahima Kirakoya:** Writing – review & editing, Validation, Supervision. **Amidou Bako:** Writing – review & editing, Validation. **Mohamed Simporé:** Writing – review & editing, Validation, Supervision, Conceptualization. **Salle Joseph Ramdé:** Writing – review & editing, Validation, Supervision, Methodology. **Sinaly Soaré:** Writing – review & editing, Visualization, Validation, Methodology. **Clotaire Alexis Marie Kiemdiba Donega Yaméogo:** Writing – review & editing, Validation, Supervision. **Adama Ouattara:** Writing – review & editing, Validation, Supervision, Methodology, Investigation, Conceptualization.

## Consent

Signed consent was obtained from the patient.

## Conflicts of interest

The authors declare that there are no conflicts of interest regarding the publication of this article.

## List of Abbreviations

ART: Antiretroviral therapy
CT: Computed tomography
DSLNB: Dynamic sentinel lymph node biopsy
HIV: Human immunodeficiency virus
HPV: Human papillomavirus
PSA: Prostate-specific antigen
SCC: Squamous cell carcinoma
